# “Older people tend to be invisible”: a qualitative study exploring the needs and inclusion of older Syrian refugees in the context of compounding crises in host country, Lebanon

**DOI:** 10.1186/s13031-022-00496-4

**Published:** 2022-11-19

**Authors:** Sarah Hachem, Souad Ali, Sarah Al-Omari, Maya Abi Chahine, Sasha Abdallah Fahme, Abla Mehio Sibai

**Affiliations:** 1 Department of Health Promotion and Community Health, Faculty of Health Sciences, American University of Beirut, Beirut, Lebanon; 2 Department of Health Behavior and Health Education, School of Public Health, University of Michigan, Ann Arbor, MI USA; 3 Department of Epidemiology and Population Health, Faculty of Health Sciences, American University of Beirut, Beirut, Lebanon; 4 Faculty of Health Sciences, American University of Beirut, Beirut, Lebanon

**Keywords:** Refugee, Older population, Aging, Humanitarian crises, Syria, Lebanon

## Abstract

**Background:**

Older Syrian refugees in Lebanon are a marginalized population with under-recognized health needs. The inclusivity of this population within the humanitarian response is poorly understood. This study aims to identify the unique needs of older Syrian refugees in the context of recent concurrent crises in Lebanon, and explore the extent to which they are being met and prioritized by local and international aid agencies.

**Methods:**

We conducted in-depth interviews with a snowball sample of 26 stakeholders from 11 organizations operating in the health, nutrition, and water, sanitation, and hygiene sectors. Data analysis followed principles of thematic analysis.

**Results:**

Concurrent political, economic, and public health crises in host country promoted income insecurity among older refugees and increased dependency on younger relatives, leading to food insecurity, neglect, and poor health outcomes, including the sequelae of untreated non-communicable diseases. Mental illness was perceived to be exacerbated by Covid-19 related challenges, including social isolation, uncertainty about the future, and additionally due to feelings of guilt related to economic dependence and fundamental exclusion from labor force participation. Despite their vulnerability, older refugees are overlooked by the humanitarian response, which may be related to a lack of data. Pervasive medication shortages in the setting of the economic collapse, as well as inaccessible physical environments and competing interests were all identified as major barriers to care.

**Conclusions:**

Older Syrian refugees in Lebanon experience dual vulnerability that is acutely exacerbated in the setting of concurrent crises. Sociopolitical, economic, and cultural barriers promote social exclusion and may confer an increased risk of income and food insecurity in this population, with significant implications for health. Humanitarian aid agencies operating in the context of fragmented, under-resourced health systems are currently unable to sufficiently address multi-faceted needs of this community. We recommend moving away from a donor-dependent model of aid by allocating resources toward strengthening inclusive national health systems that emphasize preventative care. We further call for age-disaggregation of routine data and normalization of data sharing among stakeholders in the academic and public health sectors in order to develop evidence-based initiatives that can meet the needs of this under-served community.

## Background

Population aging is one of today’s megatrends and one of humanity’s most remarkable achievements. Older persons, often defined as individuals aged 65 years and older, accounted for just 6% of the world’s population in 1990, but are expected to represent one in six people by 2050 [1]. Nearly two thirds of older people live in low- and middle-income countries (LMICs), which are disproportionately affected by armed conflict and forced displacement [2]. Governments in LMICs have limited capacity to adequately confront such crises and may depend instead on humanitarian aid organizations to meet basic population needs.

The specific needs of older populations affected by conflict and forced displacement, including health, nutrition, social protection, and water and sanitation hygiene (WASH), have been long overlooked by aid organizations, which traditionally center their response around physical injuries, acute problems, infectious diseases and maternal and child health [3–5]. For instance, the United Nations Refugee Agency (UNHCR) led a cross-sectional study of 21 refugee camps and settlements in Bangladesh, Kenya, South Sudan, Uganda, and Zimbabwe, which found age-based disparities in WASH accessibility, attributed in part to inattention to older persons’ needs in the design of WASH facilities [6]. A mixed-methods study of Burundian refugees in Tanzania and internally displaced persons in eastern Ukraine additionally found that humanitarian organizations in these settings were largely unable to address older and disabled refugees’ needs, owing to limited communication between specialty organizations which serve either elderly or disabled populations, but not both, minimal age-disaggregated data, and lack of participatory efforts to actively engage beneficiaries in the design and development of service initiatives [7].

One major barrier to inclusivity may be a lack of financial support. Though there are numerous guidelines and policies which acknowledge older people’s rights in emergency-affected contexts, this population nonetheless remains largely excluded from the humanitarian agenda and international funding [8–13]. For instance, an analysis of humanitarian financing which examined 16,221 global humanitarian initiatives implemented between 2011 and 2014 demonstrated that projects specifically targeting older persons accounted for under 1% of funded programs [14]. The investigators identified a global lack of age-disaggregated data and thus evidence of older people’s needs as a major barrier to developing and financing targeted humanitarian programs, which tended to be concentrated in the food security, health, and shelter sectors, despite the fact that older age confers cross-cutting vulnerabilities that warrant a comprehensive and holistic response [14].

Compared with other population segments, older people in crisis settings experience greater barriers to accessing even basic healthcare services. For instance, an inadequate supply of affordable medications for chronic conditions increases older refugees’ risk of interrupted treatment and adverse complications [15–17]. Furthermore, resources such as long-term care for the most vulnerable and home-bound populations who may be living alone and/or be disabled, are scarce [7]. Additionally, older refugees suffer from loneliness, self-neglect, depression, and deteriorating mental health due to low social support and fragmented social networks, especially when residing in countries with dissimilar cultures [18, 19]. Yet, there is a scarcity of mental health and social support resources in such contexts, leaving these needs largely unaddressed [20].

The exclusion of older populations from the humanitarian response may be in part related to a greater knowledge gap around their needs within settings of crisis. Findings from a systematic search of four databases and over 5000 international health journals in 2012 revealed just 13 relevant articles that discuss older people in emergencies [21]. Furthermore, at the data collection level, needs assessments and monitoring are rarely disaggregated by age, making it challenging to systematically appraise the unmet needs of older persons. Consequently, humanitarian organizations often prioritize aid to other vulnerable groups, mainly women and children, over older people [22]. The preferential assistance of certain refugee populations over others may be rooted in a ‘hierarchical’ framework, which recognizes some groups as more meritorious than others by virtue of age, gender, religion, and other aspects of identity [23–25]. Scholars argue that this hierarchy may be driven by donors’ financial interests, as their investments may be more ‘cost-effective’ when directed toward, for instance, reproductive-age women and/or younger refugees participating in the labour force [23].

These issues may be particularly relevant to older Syrian refugees. The Syrian refugee crisis, now in its eleventh year, remains among the most profound and intractable complex emergencies of our time. Nearly fourteen million individuals have been forcibly displaced, either internally or to neighboring countries, including an estimated 1–1.5 million refugees in Lebanon, which has the highest per capita population of displaced persons in the world [26], when considering the additional approximately 415,000 Palestinian refugees who reside there [27]. Yet, there are legislative barriers restricting Syrian refugees’ economic opportunities, physical and social mobility, and residence in Lebanon, thereby threatening their aid eligibility and increasing their risk of food and shelter insecurity [28, 29]. Unlike Palestinian refugees in Lebanon who access healthcare through the United Nations Relief and Works Agency, Syrian refugees are often confronted with prohibitively expensive out-of-pocket healthcare expenditures, owing to limited coverage by UNHCR in the setting of a highly fragmented and privatized health system [30]. The protracted refugee crisis has been worsened too by several years of worsening economic and political crises in Lebanon, all of which were acutely exacerbated by both the Covid-19 pandemic and the 2020 explosion of the Beirut port [31–33]. While prior to these concomitant crises, the concerted response of the Lebanon Crisis Response Plan actors had successfully mitigated a severe decline in Syrian refugees’ socioeconomic vulnerability [34], currently over 90% of Syrian refugees in Lebanon are estimated to live in extreme poverty [26]. These harsh and uncertain living conditions uniquely impact older refugees [35], who may experience social isolation and, in the setting of insufficient humanitarian aid, depend upon younger family members for survival. Such dependency upon informal caregiving may additionally prevent younger relatives from generating income, as one study found that nearly one third of Syrian refugees in Lebanon cited caring for a dependent family member as the primary reason for unemployment [26].

The degree of inclusivity of older Syrian refugees within the humanitarian response in Lebanon, particularly in the context of these recent concomitant political, economic, and public health crises, is poorly understood. Older studies done prior to the onset of these crises suggest that displaced older Syrians have substantial unmet needs requiring immediate planning and intervention [35–40]. One qualitative study, also conducted prior to the onset of these crises, similarly described a lack of purposeful and holistic programs and policies that assess and respond to older refugees’ specific concerns [41]. The current crises in Lebanon have rendered older Syrian refugees even more vulnerable. Further, their incorporation into the humanitarian response in Lebanon has not been explored in the setting of these overlapping crises. This study aims to fill this gap, by looking at the inclusion and prioritization of older Syrian refugees among humanitarian aid providers in Lebanon specifically in the context of these recent crises. We explore the attitudes, beliefs, and perspectives of humanitarian actors on: (1) the degree to which aid organizations coordinate and tailor interventions to older adults’ needs in the setting of overlapping crises; (2) the challenges which humanitarian actors may encounter when providing care for older refugees; and, (3) the impact of targeted programs, or lack thereof, on older Syrian refugees’ physical and mental health.

## Methods

### Study setting

This is a qualitative study which employed a series of in-depth interviews with stakeholders working in non-governmental organizations (NGOs) that provide services to Syrian refugees in Lebanon. These NGOs operate in geographically diverse regions of the country, though primarily in the Beirut, South, and Bekaa governorates of Lebanon, which include rural areas with a high-density population of Syrian refugees. The organizations serve both urban and rural populations of refugees, including those who reside in informal tented settlements.

### Sampling and recruitment

A detailed list of humanitarian agencies operating in Lebanon and providing services to Syrian refugees was compiled based upon a search of the grey literature as well as our team’s prior field experience. We identified 18 potential NGOs from which to recruit, and ultimately selected 11 based upon our inclusion criteria of: (1) provision of services within the health, nutrition, and water sanitation and hygiene (WASH) sectors; and (2) inclusion of older refugees in service provision, though not necessarily exclusively (i.e., organizations could cater to other age groups as well). Notably, the definition of “older adults” varied among these organizations, with some considering individuals aged 55 years or older to meet criteria, and others adopting a more traditional definition of 65 years or greater. These organizations included local, international, and UN agencies (Table 1). A formal letter which included the purpose and the detailed objective of the study was sent to high level administrative staff working in these agencies for recruitment purposes. Interested prospective participants were provided with additional details on study procedures.

**Table 1:** Characteristics of the organizations included in the study

Sector	Type			Total	Scope of work and services provided
	Local	International	UN Agency		
Health	3	2	0	5	Basic healthcare through subsidized primary healthcare services including management of acute and chronic diseases, basic laboratory tests, imaging and essential medications
					Mobile health services
					Subsidized tertiary care services
					Mental health and psychosocial support
WASH	2	0	1	3	Provision of clean water for drinking, cooking, personal hygiene and household cleaning
					Provision and maintenance of latrines
Nutrition	1	1	1	3	Implementation of nutrition programs at national, local, and community levels
					Services to treat undernutrition
					Provision of nutritious food boxes, food vouchers and hot meals
Total	6	3	2	11	

Three organizations delivered services in more than one sector and hence grand total exceeds 11

A snowball sampling approach was adopted to recruit a total of 26 participants from these 11 agencies (2–3 from each). We adopted a maximum variation approach in which we targeted a balance of high- and intermediate-level directors and managers, as well as social workers and healthcare providers such as doctors, nurses and psychologists who have direct contact with beneficiaries.

We initially interviewed directors and then asked them at the conclusion of the interview to recommend a mid-level colleague from their organization to participate in the study. This next group of interviewees were accordingly each asked to nominate two to three fieldworkers, from whom we selected one. Of the 27 prospective participants approached, only one declined the invitation.

### Data collection

In-depth interviews were conducted between June and August of 2020. Due to the ongoing Covid-19 pandemic, the majority of interviews were conducted remotely, either through a virtual platform or by telephone. Upon interviewee request, eight interviews were conducted in-person in private offices at the humanitarian agency in which the participant was employed. Personal protective equipment and appropriate distancing measures were employed to ensure public health safety during these encounters.

A semi-structured interview guide was developed to verify the consistency between interviews and help promote reliability [42]. The interview guide was designed based on the “Humanitarian Inclusion Standards for Older People and People with Disabilities”, published by the Age and Disability Consortium as part of the Age and Disability Capacity Program (ADCAP) in 2018 [43], which is now part of the Sphere Humanitarian Standards Partnership [44]. The standards are considered a positive step on the way to set the bar for the performance and accountability of humanitarian agencies working in emergencies with older people and people with disabilities. The inclusion standards tackle activities at all the phases of the response, from data collection, monitoring, programming, addressing barriers, building capacities and resilience, and programs management activities [43].

The interview guides included also open-ended questions inquiring about the perceived needs of older Syrian adults, the services provided by the NGO, the challenges related to service provision, perceived barriers to service accessibility, and recommendations to better serve older Syrian refugees. Interviews were conducted either in colloquial Arabic or in English, depending on the preference of the interviewee, to ease the freedom of expression of opinions and avoid any misapprehension related to language barriers [45].

Interviews were audio-taped with participants’ consent. Only three of the 26 declined recording; in these instances, the interviewer took notes during the interview. The mean duration for each interview was approximately 40 min. The total number of interviews was guided by data saturation.

### Data analysis

All audio recordings were transcribed verbatim and those conducted in Arabic were translated to English. Transcripts were anonymized, and coded in pairs. Data analysis was conducted iteratively and followed principles of thematic analysis [46]. All audio recordings were transcribed verbatim and those conducted in Arabic were translated to English. Transcripts were anonymized, and coded in pairs. Data analysis was conducted iteratively and followed principles of thematic analysis [46]. Using an inductive approach, two study team members independently read and analyzed the transcripts through an initial line-by-line coding. A descriptive codebook was developed by each, which was then discussed and compared within and between interviews to ensure data saturation was attained. The codes were evaluated to identify any relationships then categorized based on underlying themes, with consideration of the research aims. The salient and recurring information was discussed and a separate matrix was formed to capture and cluster emerging themes, which was reviewed by two additional study members to ensure a coherent pattern was apparent. The subthemes and overarching themes were then defined and reorganized until consensus was reached.

## Results

We identified three major themes and several sub-themes (Table 2). Below, we provide a narrative description of the social and structural determinants of perceived health needs in this population, and the extent to which they are addressed by current available resources. We then discuss the primary challenges to responding to these needs, specifically in the context of multiple, competing crises in Lebanon.

**Table 2:** Major themes and sub-themes

Theme	Sub-themes
Social and structural determinants of health in the context of concurrent crises	Lack of income and increased dependency
	Food and water insecurity
	Disability and inadequate caregiving
Older Syrian refugees’ health needs and services	Sequelae of non-communicable diseases in the setting of poor healthcare accessibility
	Mental illness related to social isolation, feelings of guilt, and uncertainty about the future
	Mobile clinics and home services, including humanitarian aid and psychosocial support
Barriers to service provision and accessibility	Limited access to medications in the setting of fragmented health systems and unsustainable models of humanitarian aid
	Barriers related to the Covid-19 pandemic
	Inaccessible physical environments

### Social and structural determinants of health in the context of concurrent crises

#### Lack of income and increased dependency on others

Often lacking independent sources of income, older Syrian refugees were perceived to have become increasingly dependent upon their relatives for survival and consequently prioritize the needs of their relatives over their own, which may lead to poor mental and physical health outcomes. Structural barriers may exacerbate income insecurity in this population. Some participants identified the lack of legal residency, which carries implications for mobility and may pose challenges related to income generation and service accessibility, as a major stressor. One participant noted that older refugees may be unable to renew their annual residency due to high costs, preferring instead to allocate these funds towards younger relatives who may potentially enter the labour force:

“[Older Syrian refugees] are not the income generators in the family. They don’t have their own money and so they don’t decide how money is spent…The percentage [of older Syrian refugees] who don’t have a valid residency…is much higher than younger age groups… Older refugees are not even going to think of renewing their documentation because obviously it is costly…they're going to renew the residency of the people who are working and who can move freely, so if they’re stopped at a checkpoint, they have a valid residency so they’re not arrested or deported.”

- Project Coordinator, Local Health NGO

Similarly, citing legislation which prohibits Syrian refugees in Lebanon from working in sectors outside of agriculture, construction, and domestic cleaning [29], some participants felt that frailty and disability may preclude older Syrian refugees from obtaining gainful employment and thus achieving financial independence:

“[Older Syrian refugees] cannot work… there a lot of [refugees] that work in agriculture, there are [refugees] that work in construction... [Older Syrian refugees] do not have the mobility, they do not have the physical strength. Usually, the occupations that the government allows... Syrian refugees to work in are occupations that require physical effort…So of course [older Syrian refugees] are prone to… [having] less income, or less sources of income.”

- Coordinator, International Humanitarian NGO

Several participants mentioned that high rates of unemployment in this population promote dependence on others. To overcome these disparities, participants noted that older Syrian refugees may resort to menial labour or begging in order to secure minimal income:

“The elderly [refugees] are being asked to go back to ‘work’, and by ‘work’ I mean selling [things] like napkins on the street or begging… we found that more and more families were asking [older Syrian refugees] to contribute to generate some kind of income alongside the rest of the family.”

- Project Coordinator, International Humanitarian NGO

“You’ll see an [older Syrian refugee] on the road, tremulous because he has Parkinson’s Disease, driving a taxi because he wants to make some money that will keep him alive.”

- Director, Local Humanitarian NGO

While the income-generating needs of older refugees were widely recognized by study participants, the majority of services offered by aid organizations nonetheless focus on vocational training for younger, working-age refugees. This focus on skill-building was perceived as cost-effective, as older populations that are unable to work require greater financial investments:

“Our biggest intervention for refugees has been skill development…. computer literacy, English classes, how to develop a CV, how to ensure they're putting themselves in the best position to secure work… I don't think anyone is specifically neglecting [older Syrian refugees] but you know, it costs more money to help the elderly…It’s easier to help a young Syrian refugee [access] skill development…Additionally, large health costs as well as…deteriorating health among elderly persons drives up their costs [of care].”

- Director, International Humanitarian NGO

Furthermore, older Syrian refugees were commonly perceived by aid providers as not prioritizing their own health due to competing needs for essentials such as food and shelter, which are increasingly difficult to secure in the setting of the country’s economic collapse. In describing the predicament that older refugees may encounter, one participant remarked:

“[We] receive feedback from the older people, especially Syrian refugees that sometimes they [have to] go to Syria to get their medications …[Sometimes] they can’t even get them from Syria. They have other priorities like rent. [They] don’t have a steady income and…they tell you: ‘The UN stopped this and that payment’ … some [older refugees] have disabilities or diseases and they don’t have money to get their medications so… it’s like compounded vulnerabilities.”

- Project Coordinator, Local Health NGO

#### Food and water insecurity

All participants identified food insecurity to be a leading issue among older Syrian refugees, particularly since the onset of the economic crisis in Lebanon. The prohibitively expensive cost of healthy food in the setting of the depreciation of the Lebanese currency was cited by participants as the major driver of food insecurity. Additional barriers to food security in this population included: a lack of a kitchen, perceived high prevalence of dementia that precludes older Syrian refugees from cooking, and physical disability which prevents older Syrian refugees from leaving their homes to purchase food. For those able to access food, the lack of available nutritious options due to financial constraints was thought to result in a limited and unhealthy diet. Access to produce, dairy, poultry, and meat was perceived as extremely limited if not altogether impossible:

“A lot of the diet is very heavy on carbohydrates. People don't have access…to protein, so it's a lot of bread, a lot of potatoes, lots of pasta, you know starches that are filling. Also, there's high intakes of sugar and also very limited options for activity. It's not like they can go for a walk in a nice neighborhood…[they] have quite a sedentary life and not a very diverse diet.”

- Director, Local Humanitarian NGO

Several participants described negative coping mechanisms among older Syrian refugees, including intentionally reducing their food intake to ensure there was enough food for their children and grandchildren. Participants noted that some older refugees had just one restricted meal per day:

“[Older Syrian refugees] eat lentils, rice, tea, and sugar… One family told me that what matters is that they have bread and yogurt for lunch. Most [older Syrian refugees] have one meal during lunch, like they drink tea with yoghurt, or they would have one meal in the morning. We do not have someone here who eats two or three meals. No, they barely have one meal”.

- Director, Local Humanitarian NGO

Similar concerns were raised around access to clean water. Recognizing that clean water is a relatively scarce commodity in Lebanon, participants discussed the worrying trend of refugees resorting to securing drinking water from often unverified and unsafe sources, especially in areas out of organizations’ reach and where municipal WASH services are not accessible.

#### Disability and caregiving

While participants perceived most older Syrian refugees to be either partially or fully dependent on informal caregivers for activities of daily living (ADLs), they noted that competing economic interests within same household, now worsened in the setting of current crises, may lead to poor care and, in some cases, neglect. Indeed, several participants expressed the need for facilities or programs to house or care for this population, citing that they may be exploited by others upon whom they may depend for basic necessities. Along these lines, respondents raised concerns about whether informal caregivers have the necessary skills to provide adequate care for older refugees with advanced comorbidities and disability.

“[Older Syrian refugees] feel like a burden. [They have] all these medical needs, and [their] families are not able [to care for them]. Though they would like to prioritize [their] medicine…but their family has other needs… [their] grandchildren have to go to work and drop out of work and drop out of school [to support them]. So, I think there are a lot of factors [contributing to social isolation]. Neglect is one of them.”

- Project Coordinator, International Humanitarian NGO

“There is nothing accessible [to older Syrian refugees]. If [they] walk on the road, there are hundreds of potholes…[Our] infrastructure is not prepared [to support their needs]. And the people that used to support them who are younger, the people they live with, they suffer in the ... vicious cycle of the economic collapse. Everyone is thinking about how they can educate their children or work… [they can] no longer give the same attention to [older Syrian refugees], who will become isolated.”

- Director, Local Health NGO

Along these lines, participants described a major need for accessible latrines in the homes of older Syrian refugees. Though only representing roughly one fifth of Syrian refugees in Lebanon [26], those living in tented settlements do not have private latrines in their homes and have to utilize those in shared spaces, thus necessitating they walk a certain distance. This is particularly hard for older or disabled refugees who have difficulties moving around, as participants noted that latrines may not be readily accessible; they may be a significant distance away and/or require ambulation up steps. Furthermore, many participants felt that, without support from caregivers, older Syrian refugees are unable to successfully navigate health systems, whether due to disability or poor health literacy:

“Older people tend to be invisible. They don't know where services are … They aren't going to come to you; you have to go to them… They have decreased mobility not just in the sense that they can't move … [but also] in the sense of knowing what programs exist that they can benefit from.”

- Manager, UN Agency

### Health needs and services

#### Sequelae of non-communicable diseases in the setting of poor healthcare accessibility

Non-communicable diseases (NCDs) such as hypertension, diabetes, and dyslipidemia were frequently noted by participants to be a major issue among older Syrian refugees in Lebanon. Stress and war trauma exposure were perceived by some to be major drivers of NCD severity, though often these diseases may go undetected, as described by one participant:

“The major diseases diagnosed [among older Syrian refugees] are related to psychological stress and high blood pressure, in addition to diabetes. Because the majority of patients feel a change in their health after being faced with strong psychological trauma, or if they were faced with missiles, torture, or the death of a close relative … With time they realize they have a significant increase in their blood sugar levels, and they do not feel it. Their blood pressure increases, and they do not know that they suffer from hypertension.”

- Director, Local Health NGO

Alluding to insufficient preventative health infrastructure in Lebanon, participants described hosting screening events in diverse communities to detect hypertension and diabetes in older populations that included refugees. Yet, diagnostic data in the absence of reliable follow-up care fails to adequately address NCD-related needs in this community, leading to what some participants describe as preventable sequelae of untreated disease:

“There was a…woman, old in age and Syrian. She was living…in a [remote] village ... When we were doing [NCD] screening in another village … we discovered that her blood pressure is very high, and that she is not taking any medications… We requested her family to bring her to our [health] center. They did not bring her. She became very sick…we noticed that the carotid arteries became very small, like [her untreated conditions were] causing some light strokes…Yet, they would never bring her… The doctor told [her son] that she is in need of a hospital because she needs scans for her head. [Her] speech is a bit affected…her movement is slow. He told him [her hypertension] might have caused light strokes… But still, [her family] were not taking her…because she is a burden. [Their response] is like: 'But from where are we going to get her medications?’”

- Nurse, International Health NGO

Indeed, hesitancy to seek care due to high costs was described not only among caregivers, but older Syrian refugees themselves:

“Most of the time [older refugees] keep silent and don't disclose their symptoms because what they say is that: ‘We are worried if we tell, our kids can't [afford] to help us so why would we worry them about something they can't control?’ And a number of people say that they have been trying to save money for two months or three or four in order to be able to do a certain test.”

- Manager, International Health NGO

Such behavior was perceived by some to be related to broader feelings of guilt among older Syrian refugees, particularly due to growing income insecurity and dependency on others.

#### Mental illness related to social isolation, feelings of guilt, and uncertainty about the future

Depression and anxiety were identified to be major health needs in this population. Several participants suggested that older Syrian refugees may struggle more deeply with depression and anxiety than do younger refugees due to feelings of helplessness and being unable to contribute meaningfully to society, which may engender hopelessness. Such feelings may be exacerbated by growing dependence on younger relatives due to their inability to generate income:

“[Older Syrian refugees] do not have the ability to adapt like the youth because … they do not have hope in tomorrow like young people … Suddenly they find themselves sitting in a tent; their neighbors treat them as second-class citizens … Honestly the health condition of elders is very related to ... depression because of the fear of tomorrow and the fear of death, in addition to societal stress.”

- Director, Local Health NGO

While such fears, which interviewees described as exacerbated by multiple crises in Lebanon, may be pervasive among vulnerable communities, participants noted that these are particularly heightened among older individuals, who may harbor a keener recognition of their own mortality, and may be less likely to seek care, due to feelings of guilt and futility, and prioritization of other more imminent needs:

“Culturally, the way [the elderly] express themselves is like: 'I'm dead anyway, these are just extra years.' We hear that a lot.”

- Manager, International Health NGO

Participants additionally perceived social isolation as a potential driver of poor mental health, particularly for older Syrian refugees who live alone. Several participants noted that the Covid-19 pandemic may evoke specific anxiety and thanatophobia in this community, describing some older refugees as being more fearful for their health and often refusing to leave their homes, especially if they had NCDs:

“[The elderly] would come to the hospital, and they would be very scared… [They’ve heard]: ‘You will probably die [of Covid-19]. Like you have to pay attention because you might die.'… [They are getting these messages from] society, from their neighbors.”

- Nurse, International Health NGO

#### Mobile clinics and home services, including humanitarian aid and psychosocial support

Several participants described the provision of either mobile clinics or home-based care for older Syrian refugees who may be dependent in their ADLs. While many of the organizations represented offer such services, these are often limited in scope and duration, and thus do not sufficiently meet beneficiaries’ needs:

“We were providing home-based care [for disabled older Syrian refugees] and a…mobile clinic…[Volunteers] would go to the houses [of disabled older Syrian refugees], [provide] physiotherapy, [follow-up] their NCD management… but there is no continuity. [When] we stopped the project, all the home-based care stopped.”

- Field Officer, International Health Organization

Several of the organizations focused on psychosocial support services, which ranged from one-on-one therapy sessions to group activity classes. Participants from two organizations reported specifically targeting older Syrian refugees with peer support groups, individual counseling, and virtual psycho-support sessions. Additionally, some offered life skills campaigns and opportunities for socializing through programs that include craft-making, sewing, and exercise. While not providing direct aid, such activities were felt by some participants to be empowering and help promote independence among older Syrian refugees:

Several of the organizations focused on psychosocial support services, which ranged from one-on-one therapy sessions to group activity classes. Participants from two organizations reported specifically targeting older Syrian refugees with peer support groups, individual counseling, and virtual psycho-support sessions. Additionally, some offered life skills campaigns and opportunities for socializing through programs that include craft-making, sewing, and exercise. While not providing direct aid, such activities were felt by some participants to be empowering and help promote independence among older Syrian refugees:

“[We provide] life skills as a means of empowering [older Syrian refugees]… the aim of these projects is to enhance their well-being and at the same time, [let them feel] that they still have something to contribute in society…There was a period of time where we offered gardening sessions and we gave gardening tool kits, so [older Syrian refugees] could go home and still have something to do, like planting seeds, or for example, loom-weaving classes … When you give the older person basic literacy, you're decreasing his dependency on the people around him.”

- Project Coordinator, Local Health NGO

Yet, such activities were perceived by some participants as insufficiently addressing this population’s essential needs:

“One time we were giving out…cooking tools like pots… [and] an older Syrian refugee woman told me ‘We don't want pots we want money and we will buy what we need.’”

- Project coordinator, Local Health NGO

To address food insecurity, humanitarian organizations do distribute food and/or food vouchers to Syrian refugees, yet these services are not tailored to older Syrian refugees’ needs. For instance, food parcels include non-perishable goods but often lack fruits, vegetables, and protein sources, and thereby may not be sufficient to meet older individuals’ required intake. However, several participants mentioned that social workers at their organization purchase and deliver food to homebound refugees, who were often older and had chronic diseases. Another described the establishment of a community kitchen to help respond to growing food insecurity.

Finally, some participants described providing resources for disabled refugees, among whom older individuals are overrepresented, such as hearing aids, wheel chairs, crutches, walkers, and beds for homebound individuals. However, these services, offered in collaboration with other organizations, were conditional on funding and therefore only available for a limited period of time.

### Barriers to service provision and accessibility

#### Fragmented health systems, unsustainable humanitarian aid, and limited access to medications

Older refugees were perceived to experience an increasing number of challenges to accessing humanitarian aid programs in Lebanon, particularly in the setting of concomitant crises. Critical shortages of essential items including medications were among the most significant challenges identified in this study, with multiple participants noting that older Syrian refugees may return to Syria in order to obtain medications, a trend that has been previously been described [30]. While cost barriers have long impacted refugees’ ability to secure medications [47], participants noted that this has been acutely exacerbated by the recent crises in Lebanon. Following a decision by the Lebanese government to lift states subsidies on medications, 85% of which are imported [48], pharmacies, hospitals and humanitarian aid organizations have been facing difficulty in securing chronic medications:

“We are a primary care NGO and we work with the government…We get medications and vaccines from the Ministry of Health and we give them to the people. What happened? There's no more. I spoke to the Ministry of Health saying ‘I want a vaccine’ they said there isn't any. I said I want chronic medication and they said: ‘There is none figure it out’. What do they mean ‘figure it out’? This is a challenge…If somebody has diabetes or hypertension, [the government’ response is]: ‘Sorry he can't get it, just figure it out.’… I think we are seeing the downfall of the government and the rise of the NGOs just like in a war.”

- Director, Local Health NGO

Unreliable access to medications was felt by participants to be a significant barrier in the broader context of interrupted service provision owing to lack of funding. For instance, a number of home-based services which provided essential care for disabled refugees and psychosocial support programs were discontinued despite their popularity and success in the communities served:

“The psychosocial support activities [we provide] are great…the classes and support groups that older people attend, they discuss a lot of things, and their feedback is very positive. They improve, they listen to each other, and they share the feelings of loneliness, they... share their experiences in their country, in Syria with the Lebanese …but there is no continuity… Once we finish our funds with our partner [organization]…we can no longer continue.”

- Field Officer, International Health NGO

#### Barriers related to the Covid-19 pandemic

The Covid-19 pandemic was perceived to have worsened or created additional barriers to service accessibility among older Syrian refugees in Lebanon. Firstly, already-minimal home care services were further diminished; participants indicated that with the implementation of nation-wide lockdown measures, very few people were sent to provide services to homebound older refugees:

“In the era of Covid-19 there have been big challenges. Very few people will go into the homes and provide care [to older Syrian refugees]. And I think it's also because people are not [compensated] enough to support, to have the protective gears that they need to feel safe.”

- Director, Local NGO

Participants noted that the pandemic similarly had significant implications on communication and community outreach efforts for older refugees in particular. Some felt that the increased use of virtual platforms such as WhatsApp led to lower retention-in-care levels broadly speaking among refugees, who tend to be mobile and often change their telephone numbers. Medical doctors and social workers interviewed expressed concerns, noting that in the setting of social distancing measures, communication over WhatsApp and other electronic platforms tended to exclude the majority of older Syrian refugees who do not have access to mobile phones and/or lack digital literacy:

“[Older Syrian refugees] don't own mobile phones and some of them cannot afford having mobile data or internet. Their caregiver at home or other family members are the ones who have phones…We have created groups and added [family members] but some of them left the groups because they were bombarded with the messages.”

- Project Coordinator, Local NGO

Only one organization specifically targeted older Syrian refugees in their Covid-19 awareness and prevention campaigns. Participants from this organization indicated that the messages they disseminated were simple, direct, and easy to understand. In addition to providing brochures, information regarding the pandemic was given verbally during home visits or through WhatsApp voice notes.

#### Inaccessible physical environments

Participants across multiple organizations noted that the poor transportation infrastructure in Lebanon is a major barrier to service accessibility for older Syrian refugees. In addition to the perceived high costs and hazardous conditions of the limited public transportation options, poorly constructed sidewalks were also noted to be unfit to accommodate wheelchairs or facilitate the mobility of disabled individuals:

“There is no public transport. Public transport is either too costly or inefficient, and it’s possible that the services are in another community and not in the one you're targeting… It's a movement problem, it's a physical problem essentially.”

- Country Director, International NGO

Efforts were made across all of the organizations represented to improve the accessibility of their community centers, for instance, by installing ramps, offering wheelchairs, and ensuring elevator access. One participant also said they had staff at several areas within the center to direct and assist older people. Another participant described adding aluminum rails to assist those walking on stairs, increasing the size and number of signs throughout the building to make them easier to read, and installing a rail in the bathroom to aid with transferring from the toilet. Yet, inaccessible conditions outside of these centers may nonetheless still be a major barrier:

“We still have the big challenge that Lebanon and its facilities are, generally speaking, not old-age friendly and not disability friendly. It's not like when you go to France for example, in public transportation there are designated areas for older people and pregnant women, and there are ramps everywhere. We don't have this in Lebanon, which is a huge challenge.”

- Project Coordinator, Local NGO

## Discussion

The Syrian refugee crisis in Lebanon is occurring in the context of concurrent political, economic, and public health emergencies, which confer compounding vulnerabilities onto older Syrian refugees. In particular, Lebanon’s financial collapse, characterized by an estimated 90% depreciation of the currency and described by the World Bank as among the three worst global economic crises since the mid-nineteenth century [33], is perceived to create increasing dependency among refugees upon humanitarian aid for survival. Yet, the degree to which older refugees, who have historically been excluded from the humanitarian response, are prioritized in the setting of these concomitant crises has not yet been explored. This study aimed to fill this gap, by exploring the attitudes, beliefs, and perspectives of stakeholders uniquely positioned to speak to older refugees’ perceived needs and the services available to them.

We found that crises in Lebanon have severely diminished refugees’ purchasing power, with significant and synergistic repercussions in virtually every sector. For instance, limited access to clean drinking water and insufficient healthcare resources both increase refugees’ risk of water-borne diseases, which can be observed in the recently declared and ongoing cholera epidemic in Lebanon that is disproportionately impacting Syrian refugees [49]. Worsening income and food insecurity among older Syrian refugees may heighten financial dependence upon relatives for basic survival, with implications on older refugees’ health-related decision-making power. Because healthcare costs tend to be higher in this population, which has a higher prevalence of chronic illness and disability, this lack of agency may contribute to poor health outcomes, as older refugees employ negative coping strategies such as rationing of medications and food. These findings are consistent with other studies conducted among older Syrian refugees, which have repeatedly demonstrated high healthcare costs to be a major barrier to adequate control of NCDs [50, 51]. A recent cross-sectional study of older Syrian refugees in Lebanon similarly found that, in the absence of cash assistance, older refugees were more likely to prioritize more imminently essential needs such as food and water over NCD-related healthcare [50].

We additionally demonstrate that financial dependency was perceived to generate feelings of guilt and isolation among older refugees, which promote poor mental health outcomes including depression. While previous studies of this population found that older Syrian refugees who participated in domestic work such as child care did not report burdensome feelings of guilt [40], such findings pre-date recent crises and therefore may no longer be true in the current context of near-ubiquitous extreme poverty. In concordance with the literature on older refugees [7], our findings suggest that isolation may be related both to the rendering of older refugees as obsolete and passive recipients of aid, which may elicit shame, as well as to physical impediments from inaccessible environments that preclude older refugees from leaving their homes.

Furthermore, we show that the fragile and fragmented health systems, which have historically prioritized tertiary and privatized care and which have been heavily burdened by both the pandemic and the economic crisis [30, 52], as well as nation-wide shortages of medications, have led to a near-complete dependency among older Syrian refugees upon humanitarian aid for basic medical care. Such aid is often unsustainable, as many programs are temporary and contingent upon donor-based funding. Indeed, many of the activities offered to older refugees, while providing social support, do not sufficiently address underlying health determinants. This may be related to agenda-setting; often, services and programs designed for older refugees are determined by organizations and funders, rather than the communities served [23].

While this study focuses on older Syrian refugees in Lebanon, many of the findings are applicable to forcibly displaced populations in other settings. For instance, needs assessments of older refugees fleeing Ukraine have shown that over one quarter have an unmet need for medications to manage a chronic illness [53, 54]. Indeed, the higher prevalence of NCDs among older populations, coupled with ageist stereotypes of older refugees as dependent and passive recipients of aid, rather than active individuals with agency over their health and other essential needs, has long contributed to the marginalization of older populations within the humanitarian response in diverse settings [55].

**Fig. 1:**
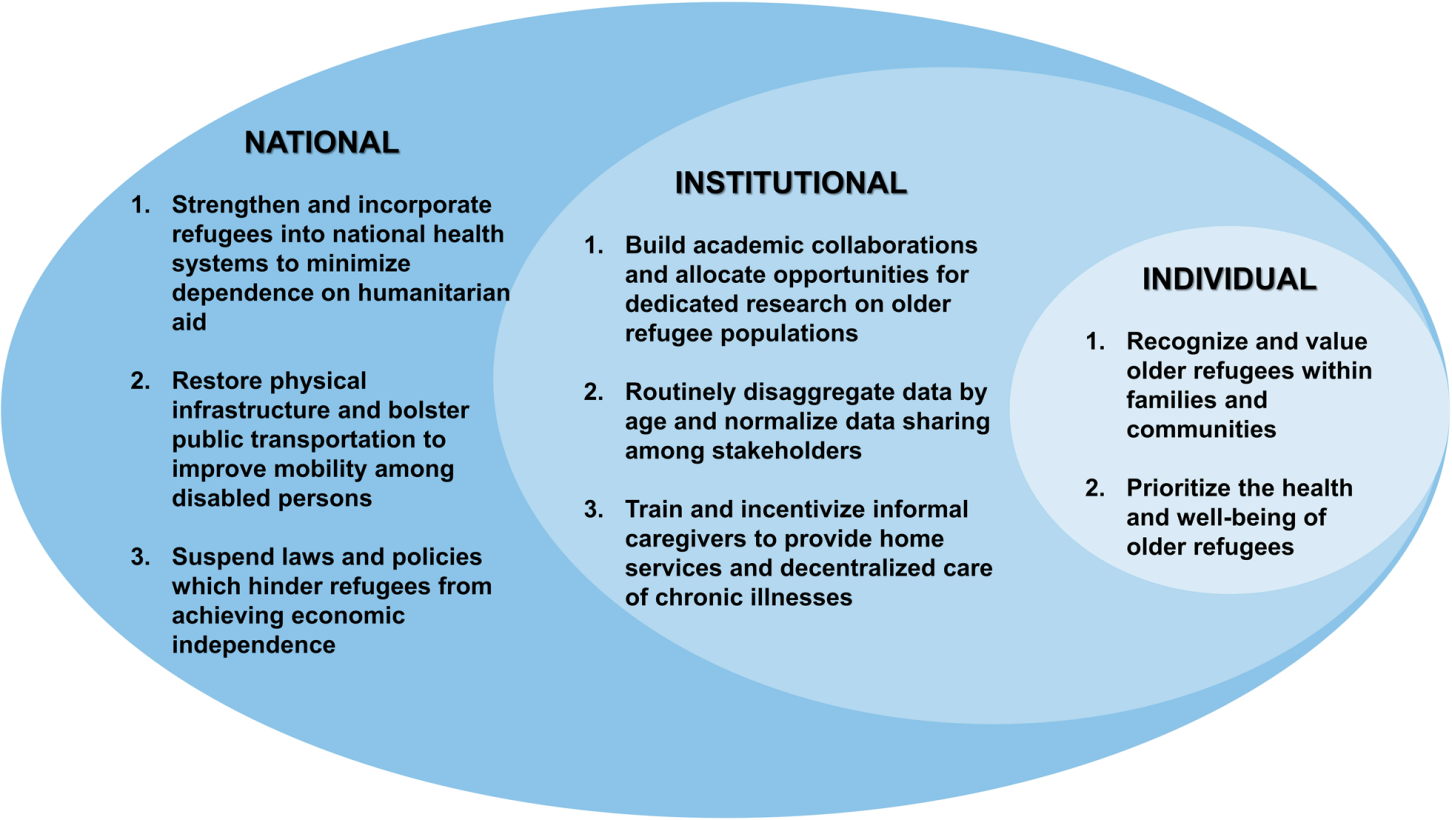
Recommendations to improve the health and well-being of older Syrian refugees in Lebanon

Based on the health determinants, needs, and barriers to care occurring in the context of compounding crises identified in this study, as well as a review of the literature, we propose the following recommendations (Fig. 1) to improve the health and well-being of older refugees living in resource-limited contexts. We consider each of these recommendations by level.

### National recommendations

The vast majority of displaced populations globally, including Syrian refugees, are settled in cities within LMICs, living within and yet segregated from host communities [56, 57]. The establishment of parallel health systems for refugees has been shown to be impractical in the setting of protracted crises [30, 58]. There is compelling evidence from diverse settings throughout the Global South that the influx of refugees has had positive impacts on host countries’ national health systems and, by extension, the health of host populations [58]. Our study findings suggest that older Syrian refugees in Lebanon may be disproportionately burdened by the sequelae of NCDs due in part to poor access to primary health services, reflecting the fragmentation and privatization of the Lebanese health system [30]. This is supported, too, by prior studies showing a high prevalence of hypertension, diabetes mellitus, and coronary disease among older Syrian refugees in Lebanon, Jordan, and Turkey [16, 40]. Study participants perceived healthcare accessibility among older Syrian refugees to further decline in the setting of growing poverty and limitations in services provided by aid organizations, which are largely donor-dependent and thereby unsustainable. Appropriating resources towards developing a unified and subsidized primary healthcare network inclusive of both host and refugee populations would diminish dependency on donor-based aid organizations, build local capacity, address shortages of critical medications, and mitigate health disparities among vulnerable refugees, including older Syrian refugees.

An essential component of healthcare accessibility, particularly for older populations, is physical accessibility of health centers. Attention to road infrastructure and provision of low-cost public transportation options were identified by participants as critical to improving healthcare accessibility for persons with physical impairment, which overwhelmingly include older populations and refugees who may have sustained traumatic injuries of war [59]. This recommendation is supported by studies demonstrating that nearly half of older Syrian refugees in Lebanon have a physical impairment that limits their ambulation [40].

Socioeconomic and political determinants of health, in addition to healthcare accessibility, must be addressed to achieve health equity for older refugees [60]. We identified income insecurity among older Syrian refugees to be perceived as a major determinant of poor mental and physical health outcomes in this community. Legislative barriers to legal residency and gainful employment must be overturned in order to increase labour force participation and address growing poverty [26, 29]. Doing so would not only mitigate food insecurity and improve refugee health, but may also improve the health of host populations, who have endured reductions in their healthcare workforce capacity since the onset of these humanitarian crises [61].

### Institutional recommendations

Though the vulnerability of older refugees in humanitarian contexts is widely recognized, older refugees’ needs remain largely underrepresented in humanitarian aid programming. Our findings suggest that this gap may in part be related to the paucity of age-disaggregated data and limited evidence on older Syrian refugees’ health needs. Indeed, one participant cited the lack of academic expertise in this field as a significant barrier to agenda-setting. Though a number of studies have highlighted the vulnerability of this population and need for targeted interventions [40, 62, 63], poor vital registration systems in Lebanon and minimal data-sharing between aid organizations perpetuate knowledge gaps and promote exclusionary initiatives, as donors may be less likely to fund projects which are not data-driven. Indeed, the lack of open-access disaggregated data in Arab countries is a well-described barrier in the public health response to other crises, including the Covid-19 and HIV pandemics [64, 65]. We previously have argued for enhanced data accessibility by institutional training on data de-identification and promoting academic-public practice partnerships [64]. Such recommendations are supported by our current study findings and are acutely needed for older Syrian refugees in particular. Institutions should apply this evidence when developing targeted vocational programs, which may empower older Syrian refugees and address multiple health determinants such as income insecurity and social isolation.

Study participants additionally described the need for home health services and long-term care for many older Syrian refugees who have limited mobility. Indeed, previous studies of this population identified 10% of older Syrian refugees in Lebanon to be home-bound, and 4% as unable to rise from bed [40]. Participants perceived that these older Syrian refugees in particular are under-served, as relatives providing informal caregiving may not have the needed skills and may have competing interests in the current socioeconomic climate which preclude them from providing adequate care. To address this gap, we recommend that humanitarian aid organizations train and incentivize informal caregivers as a means of task-shifting and decentralizing care of chronic illnesses. Such an approach has been well-described for mental healthcare provision in resource-limited settings [66], which may be particularly impactful in the Middle East, a region boasting the world’s lowest per capita availability of mental healthcare services [67]. Task-sharing interventions in low- and middle-income countries have similarly been shown to be effective at reducing systolic and diastolic blood pressure [68], and lowering low-density lipoprotein cholesterol [69], though further studies are needed to ascertain their role in managing diabetes mellitus [70]. Training community health workers to care for dependent older populations has been demonstrated to be both feasible and effective in other contexts [71], and should be explored as a cost-effective intervention among older Syrian refugees in Lebanon. Further, as informal caregiving is overwhelmingly gendered both in the Arab region and globally [72], training and compensating caregivers would additionally address economic gender disparities in labour force participation [26, 73].

### Individual recommendations

While ageing in Arab countries has traditionally been characterized by intergenerational support systems in the setting of cohesive, extended-family households [74], protracted conflicts and harsh living conditions in displacement may distort familial relationships and dependency patterns [75]. Participants alluded to such changes by expressing concerns about the recognition and, relatedly, prioritization of the health and well-being of older Syrian refugees within households and communities, i.e., among Syrian refugees. This was suggested, for example, when discussing messaging around the Covid-19 pandemic, which some participants felt dismissed by characterizing it as an issue exclusive to older populations. Participants additionally described a lack of time, skills, and attention among relatives of older Syrian refugees to provide adequate care, leading potentially to progression of chronic diseases and in some cases, neglect. As described in detail above, gaps in caregiving may in large part be due to increasingly precarious availability of resources including nutritious foods, medications, and accessible environments. However, this may also reflect larger sociocultural transformations following the destruction of family structures, properties, and assets in Syria, which may disrupt intergenerational cohesion [75, 76]. The prioritization of older refugees’ health and well-being within households and communities is critical, and rooted in others’ recognition of and respect for their added value to their communities. We believe that, the introduction of structural reforms to address older Syrian refugees’ needs, such as income and food security, home services, and accessible physical environments, as well as capacity building and compensation of caregivers, may help reduce dependency and restore intergenerational cohesion within both refugee and host population households.

### Study limitations

The study findings need to be considered in light of its limitations. A number of the sub-themes identified are not unique to older Syrian refugees. Indeed, much of the data reflected participants’ perspectives of Syrian refugee populations broadly despite targeted questions, perhaps indicative of the knowledge gap even among aid providers of this population. Similarly, some responses were not unique to refugees and encompassed older Lebanese populations, despite specific questions and probing to differentiate between older Syrian refugees and older Lebanese populations. Additionally, older Syrian refugees were not targeted in this study, limiting our interpretation of certain sub-themes, particularly those related to perceived feelings of guilt and social isolation. A separate ongoing study engaging older Syrian refugees aims to further elucidate these potential drivers of mental illness as well as other health determinants and barriers to care identified in this research.

## Conclusion

The purpose of this qualitative study was to explore stakeholder perspectives of the health needs and services of older Syrian refugees in the context of concurrent political, economic, and public health crises in Lebanon. We show that aid workers perceive legislative, economic, and cultural barriers to employment, income- and food security among older Syrian refugees as contributing to poor health outcomes in this population, including sequelae of uncontrolled NCDs and mental illness related to feelings of guilt and uncertainty. We recommend resources be allocated toward inclusive, national health systems that emphasize primary prevention of disease, rather than a donor economy which perpetuates dependency upon unsustainable models of care. Additionally, addressing income and food insecurity, prioritizing research on older populations through collaborative partnerships with academic institutions, and training and incentivizing informal caregivers to provide home-based services and care of chronic diseases, may improve the health and wellbeing of this vulnerable and overlooked population.

## Data Availability

Data sharing is not applicable to this article as no datasets were generated or analyzed during the current study.

## Abbreviations

ADLs: Activities of daily living
LMICs: Low- and middle-income countries
NCDs: Non-communicable diseases
NGOs: Non-governmental organizations
WASH: Water, sanitation, and hygiene
ADCAP: Age and Disability Consortium as Part of the Age and Disability Capacity Program
